# GAMES ARE FOR EVERYONE: A PILOT PROJECT TO FACILITATE INTERGENERATIONAL ENGAGEMENT THROUGH GAMES

**DOI:** 10.1093/geroni/igae098.0702

**Published:** 2024-12-31

**Authors:** Elizabeth Rickenbach, Loretta Brady, Adeline Clifford, Seana Gamache

**Affiliations:** Saint Anselm College, Manchester, New Hampshire, United States; Saint Anselm College, Manchester, New Hampshire, United States; Saint Anselm College, Manchester, New Hampshire, United States; Saint Anselm College, Manchester, New Hampshire, United States

## Abstract

Undergraduate students and faculty participated in a pilot project to facilitate intergenerational engagement with older adults in the community through games. Each age group shared “their generation’s” games and learned games together by playing new games curated by a game designer that are specific to an intergenerational audience. Previous research has demonstrated that intergenerational activities are beneficial to physical and mental health, mood, age-related biases, and reduced loneliness. Intergenerational activities that involve games can add an element of play, collaboration, and cognitive enrichment across all participants that may extend the benefits across all participants and encourage co-learning. Intergenerational research has often included life-interviews (older teaching younger) and when games are considered, they are often video games (younger teaching older); this project is novel in that it includes socialization, play, and cognitive engagement with traditional games with an “equal playing field” (both younger and older adults will teach and learn games). The specific purpose of the project was to demonstrate the feasibility of a facilitated game collaboration as an intergenerational activity through participant feedback. Results showed that the experience provided collaboration, joy, learning, and an opportunity for facilitated exposure to challenging topics (e.g., political discourse across generations, sharing life stories during play, expressing grief and loss during or prompted by play, experiencing frustration and satisfaction due to play experiences). Students reported that the games provide an opportunity for engagement with older adults that felt comfortable, and at times, the experience was “more about the conversation, and less about the games.”

